# Regulatory T Cells Accumulate in the Lung Allergic Inflammation and Efficiently Suppress T-Cell Proliferation but Not Th2 Cytokine Production

**DOI:** 10.1155/2012/721817

**Published:** 2011-11-15

**Authors:** Lucas Faustino, Daniel Mucida, Alexandre Castro Keller, Jocelyne Demengeot, Karina Bortoluci, Luiz Roberto Sardinha, Maisa Carla Takenaka, Alexandre Salgado Basso, Ana Maria Caetano Faria, Momtchilo Russo

**Affiliations:** ^1^Departamento de Imunologia, Instituto de Ciências Biomédicas, Universidade de São Paulo, 05508-000 São Paulo, SP, Brazil; ^2^Laboratory of Mucosal Immunology, The Rockefeller University, New York, NY 10065, USA; ^3^Departamento de Microbiologia, Imunologia e Parasitologia, Universidade Federal de São Paulo, 04023-900 São Paulo, SP, Brazil; ^4^Instituto Gulbenkian de Ciência, 2780-901 Oeiras, Portugal; ^5^Departamento de Ciências Biológicas, Campus Diadema e Centro de Terapia Celular e Molecular (CTCMol), Universidade Federal de São Paulo, 04044-010 São Paulo, SP, Brazil; ^6^Instituto Israelita de Ensino e Pesquisa Albert Einstein, 05652-900 São Paulo, SP, Brazil; ^7^Departamento de Bioquímica e Imunologia, Universidade Federal de Minas Gerais, 31270-901 Belo Horizonte, MG, Brazil

## Abstract

Foxp3^+^CD25^+^CD4^+^ regulatory T cells are vital for peripheral tolerance and control of tissue inflammation. In this study, we characterized the phenotype and monitored the migration and activity of regulatory T cells present in the airways of allergic or tolerant mice after allergen challenge. To induce lung allergic inflammation, mice were sensitized twice with ovalbumin/aluminum hydroxide gel and challenged twice with intranasal ovalbumin. Tolerance was induced by oral administration of ovalbumin for 5 consecutive days prior to OVA sensitization and challenge. We detected regulatory T cells (Foxp3^+^CD25^+^CD4^+^ T cells) in the airways of allergic and tolerant mice; however, the number of regulatory T cells was more than 40-fold higher in allergic mice than in tolerant mice. Lung regulatory T cells expressed an effector/memory phenotype (CCR4^high^CD62L^low^CD44^high^CD54^high^CD69^+^) that distinguished them from naive regulatory T cells (CCR4^int^CD62L^high^CD44^int^CD54^int^CD69^−^). These regulatory T cells efficiently suppressed pulmonary T-cell proliferation but not Th2 cytokine production.

## 1. Introduction

Regulatory T (Treg) cells have been implicated in the mechanisms that govern peripheral dominant tolerance. From autoimmunity, transplantation, and cancer to mucosal tolerance, the presence of functional Treg cells, either thymus-derived naturally occurring or peripherally-induced adaptive Treg cells have been associated with the control of inflammation [1]. 

Allergic asthma is a chronic inflammatory disease characterized by airway eosinophilia, airway hyperreactivity (AHR), mucous hypersecretion, and high titers of IgE [2]. In asthmatic patients, CD4^+^ T lymphocytes upon allergen challenge secrete type-2 cytokines such as IL-4, IL-5, IL-9, and IL-13 that in turn mediate the Th2-associated inflammatory network and IgE production [3]. It has been suggested that insufficient immune regulation by Treg cells might lead to aberrant Th2 response [4–7]. Conversely, mucosal exposure to nonpathogenic antigens results in a state of hyporesponsiveness, known as mucosal tolerance that efficiently inhibit pulmonary and systemic Th2-mediated response [8–12].

Different subtypes of regulatory T cells or suppressive cytokines have increasingly been defined as important in mediating T-cell unresponsiveness by mucosal tolerance [9, 13–15]. For instance, TGF-*β*-producing Th3 cells and IL-10-producing Tr1 cells were proposed to mediate oral and nasal tolerances, respectively [9, 16, 17]. Other Treg cells involved in mucosal tolerance have been characterized as CD4^+^CD25^+^CD45RB^low^ T cells that also express glucocorticoid-induced TNF receptor (GITR), CTLA-4, and Foxp3 [13, 14, 18–23]. 

The involvement of Treg cells in the control of allergic responses was clearly established in double T/B transgenic mice [7], a mice that harbor monoclonal CD4^+^ T-cell population specific to OVA and monoclonal B cells specific to hemagglutinin A (HA). These animals when devoid of natural Treg cells develop hyper-IgE response upon OVA-HA sensitization and challenge [7]. Previously, we have shown that oral tolerance induced by OVA feeding prevented the development of hyper-IgE production and asthma-like responses in these animals [24]. We found that oral OVA exposure induced the development of adaptive OVA-specific Treg cells that displayed suppressive activity *in vivo* and *in vitro* in a TGF*β*-dependent manner [24] indicating that Tregs are quite efficient in preventing priming of naive T cells. 

Natural or adaptive Treg cells can be further characterized as naive or effector Treg cells by the expression of chemokine receptors and adhesion molecules responsible for their preferential localization in lymph nodes or in inflamed tissues [25]. The suppressive effect of Treg cells in lymph nodes is well documented, whereas their role at sites of allergen challenge is still elusive. It has been reported that the resolution of allergic airway disease induced by long-term allergen challenge (inhalational tolerance) is associated with local accumulation of Treg cells [26]. Previous studies that employed oral or nasal tolerance to suppress OVA-induced allergic lung disease did not investigate the migration of Treg cells to the lung [23, 24]. 

In the present work, using the murine OVA model of asthma-like responses, we investigated whether Treg cells migrate to the site of allergen challenge in allergic mice or in mice made tolerant by OVA feeding before sensitization (oral tolerance). Because we found that Foxp3^+^ Treg cells as well as Th2 inflammatory cells and high levels of suppressive cytokines accumulated in the airways of allergic but not in tolerant mice, we further characterized the phenotype of these Treg cells. Upon allergen challenge, Treg cells accumulated into airways of allergic mice and showed upregulation of the chemokine receptor CCR4 and substantially downregulation L-selectin. These two surface markers could, at least, distinguish Treg cells present in the airways (CCR4^high^CD62L^low^) from those present in the draining lymph nodes (CCR4^int^CD62L^high^). In addition, airway Treg cells also upregulated molecules associated with effector/memory T cells such as CD54, CD44, and others [27, 28]. Interestingly, the increased frequency of Foxp3^+^ Treg cells in the allergic lung expressed CD69, whereas the majority of lung Treg cells from tolerant mice were Foxp3^+^CD69-negative. Finally, airway CD4^+^CD25^+^ Treg-like cells from allergic mice exhibited strong and efficient antiproliferative activity on lung CD4^+^CD25^−^ T cells but were unable to suppress type 2 cytokine production. Indeed, experiments with highly purified green fluorescent Foxp3 Treg cells confirmed the inability of these cells to suppress cytokine production by Th2 cells.

## 2. Materials and Methods

### 2.1. Mice

Female BALB/c and C57BL/6 mice at 8–12-week old, housed under specific pathogen-free conditions at the Department of Immunology, Biomedical Science Institute, University of São Paulo, Brazil, were used throughout the experiments. Foxp3-green fluorescence protein knockin (*Foxp3gfp.*KI) mice were already described elsewhere [29]; these animals were kindly provided by Howard L. Weiner (Center for Neurologic Diseases, Brigham and Women's Hospital, Harvard Medical School) and were bred at the Department of Microbiology, Immunology and Parasitology of Federal University of São Paulo. Mice were treated according to Animal Welfare guidelines of the Biomedical Science Institute (ICB-USP). 

### 2.2. OVA Sensitization and Airway Challenge

Mice were sensitized and boosted by subcutaneous route with 4 *μ*g chicken OVA/1.6 mg of aluminum hydroxide gel in 0.2 mL of sterile PBS at days 0 and 7. For the induction of airway inflammation, mice receive two intranasal (i.n.) challenges with 10 *μ*g OVA in 40 *μ*L of sterile PBS at days 14 and 21. Experiments were performed 24 h after the last i.n. OVA challenge (day 22).

### 2.3. Oral Tolerance Induction

Oral tolerance to OVA was induced by spontaneous intake of 1% OVA (grade V, Sigma-Aldrich, St. Louis, Mo USA) solution dissolved in sterile drinking water for 5 consecutive days before sensitization as previously described [24]. 

### 2.4. Bronchoalveolar Lavage (BAL)

Mice were deeply anesthetized, trachea was cannulated, and lungs were rinsed with 1.0 mL of cold PBS. Total and differential cell counts of BAL fluid were determined by hemocytometer and cytospin preparation stained with Instant-Prov (Newprov, Brazil). 

### 2.5. Determination of Respiratory Pattern

Respiratory pattern was determined before and after increasing doses of inhaled methacholine (3, 6, 12, and 25 mg/mL) in conscious unrestrained mice using whole-body plethysmograph (Buxco Electronics Inc. Wilmington, NC, USA) as previously described [12, 30]. The enhanced pause (Penh), a dimensionless value that takes into account box pressure recorded during inspiration and expiration and the timing comparison of early and late expiration was used to define the respiratory pattern. 

### 2.6. Flow Cytometry Analysis

Single cell suspensions were preincubated with FcBlock for 10 min at room temperature (BD PharMingen, San Diego, Calif, USA). Cells were then incubated in staining buffer (PBS containing 2% fetal calf serum and 0.1% NaN_3_) for 30 min at 4°C with the antibody cocktails. Samples were analyzed in FACSCalibur or FACSCanto II instruments (Becton Dickinson, San Diego, Calif, USA). Anti-mouse CD4-FITC, CD4-PerCP, CD4-Pacific Blue, CD25-PerCP-Cy5.5, CD25-FITC, CD62L-PE, CD69-FITC, CTLA-4-PE, GITR-PE, IgG2a^*κ*^-PE, IgG2a^*κ*^-FITC, IL-10-PE, IL-5-PE, and streptavidin-PE-Cy5 were purchased from BD Pharmingen (San Diego, Calif, USA). Anti-mouse Foxp3-APC and Foxp3-FITC antibodies were purchased from e-Biosciences (San Diego, Calif, USA). Affinity-purified biotinylated goat anti-TGF-*β*-bound precursor cytokine latency-associated peptide (LAP) polyclonal antibodies were purchased from R&D Systems (Minneapolis, Minn, USA). The remaining antibodies CCR4-APC, CD44-PE, CD54-PE, and CCR7-PerCP-Cy5.5 were purchased from BioLegend (San Diego, Calif, USA). 

### 2.7. Intracellular Staining for Foxp3, CTLA-4, and Cytokines

After stimulation with 2 *μ*g/mL anti-CD3 for 8 h in the presence of Monensin (Sigma-Aldrich) at 37°C, cells were first surface stained and then permeabilized for 30 min with Cytofix/Cytoperm kit (BD Pharmingen). After washing, cells were stained with anti-IL-10 and IL-5 antibodies for 45 min at 4°C. For Foxp3 and CTLA-4 intracellular staining, an additional permeabilization was performed using a Foxp3 Staining Buffer Set (eBiosience) for 30 min at 4°C. Samples were analyzed in a FACSCalibur or FACSCanto II instruments (Becton Dickinson, San Diego, Calif, USA).

### 2.8. Lung Digestion and Cell Sorting

After bronchoalveolar lavage, pieces of lung tissue were digested with collagenase (2 mg/mL) and DNase (1 mg/mL) (Sigma-Aldrich) at 37°C for 30 min. Lung CD4^+^CD25^−^ and CD4^+^CD25^+^ cells were isolated using magnetic cell sorting (Miltenyi Biotec). First, CD4^+^ cells were negatively isolated using MicroBeads to MHCII, CD8a, and B220 (Miltenyi Biotec). Negative cells were then magnetically labeled to CD25 and isolated CD4^+^CD25^−^ (>95%) and CD4^+^CD25^+^ (>90%) cells assessed by flow cytometry. In selected experiments, lung cells from allergic *Foxp3gfp.*KI mice were staining for CD4-Pacific Blue and sorted into CD4^+^Foxp3-GFP^−^ and CD4^+^Foxp3-GFP^+^ using a FACSAria cell sorter (Becton Dickinson). 

### 2.9. *In Vitro* Suppression Assay

The suppression assay was performed with CD4^+^CD25^+^ cells purified by magnetic sorting or with highly purified FACS-sorted CD4^+^ Foxp3-GFP^+^ obtained from *Foxp3gfp.*KI mice. For this, CD4^+^CD25^−^ and CD4^+^CD25^+^ cells were purified using magnetic sorting. Proliferation assays were set up in 96-well round-bottom plates and contained, per well, 2 × 10^4^ responder cells (CD4^+^CD25^−^ cells from sensitized and challenged BALB/c mice), 4 × 10^4^ APCs (Mitomycin C-treated spleen cells from TCR*αβ*-deficient BALB/c mice or from *nude *mice), and anti-CD3 (145-2C11) antibody at a 1 *μ*g/mL. Cells were cocultured at CD25^−^/CD25^+^ ratios of 1 : 1, 1 : 0.3, and 1 : 0.1. Proliferation was determined by adding ^3^H-thymidine on the third day of culture and determining incorporation 6 h later. Suppression assay with CD4^+^ Foxp3-GFP^+^ was performed with lung CD4^+^ Foxp3-GFP^+^ or Foxp3-GFP^−^ T cells that were FACS-sorted from allergic *Foxp3gfp.*KI mice. Responder cells (CD4^+^Foxp3-GFP^−^) were labeled with 5 *μ*M of *Cell Proliferation Dye eFluor-670* (eBiosciences, San Diego, Calif, USA) according to the manufacturer's recommendations. Dye labeled CD4^+^Foxp3-GFP^−^ T cells (2 × 10^5^) were than cultured without or with CD4^+^Foxp3-GFP^+^ Treg cells at ratios of 1 : 1, 1 : 0.3, and 1 : 0.1 in the presence of 4 × 10^5^ APCs (spleen cells from RAG^−/−^ mice) and anti-CD3 (1 *μ*g/mL) for 72 h. The proliferation was determined by reduction of the fluorescence intensity of Dye eFluor-670 using a flow cytometry instruments. For analysis of IL-4 and IL-5 production, responder cells (2 × 10^4^ CD4^+^Foxp3-GFP^−^) were cocultured without or with CD4^+^Foxp3-GFP^+^ Treg cells in the presence of 35 Gy-irradiated lung MHCII^+^ MACS-purified cells (4 × 10^4^) from *Foxp3gfp.*KI mice and anti-CD3 (1 *μ*g/mL). Cytokine concentrations were quantified by sandwich kit ELISA according to the manufacturer's recommendations as previously described [8].

### 2.10. Determination of OVA-Specific IgE and IgG1 Antibodies

OVA-specific antibodies were assayed by sandwich ELISA as previously described [8]. For OVA-specific IgE determinations, plates were coated overnight at 4°C with 2 *μ*g/mL of goat anti-mouse IgE antibody (Southern Biotechnology). Serum samples were added followed by addition of biotin-labeled OVA. Bound OVA-biotin was revealed by Streptavidin Peroxidase conjugate (Sigma) as previously described [8]. Hyperimmune serum from OVA/Alum-immunized BALB/c mice was used as IgE standard and arbitrarily assigned as 10.000 U/mL. For OVA-specific IgG1 antibodies, serum samples were plated on 96 wells previously coated with OVA (2 *μ*g/well). The bound antibodies were revealed with goat anti-mouse IgG1 followed by peroxidase-labelled rabbit anti-goat antibodies (all from Southern Biotechnology). The concentration of OVA-specific antibody was estimated by comparison with IgG1 standards run in parallel as previously described [8].

### 2.11. Cytokine Determinations

The levels of IL-4, IL-5, IL-10, IL-13, and TGF-*β* in the BAL fluid or supernatants from lung cells culture were assessed by a sandwich kit ELISA according to the manufacturer's recommendations as previously described [8]. Values are expressed as pg/mL deduced from standards run in parallel with recombinant cytokines. Purified and biotinylated antibodies to IL-4, IL-5, and IL-10 kits were from BD OptEIA, San Diego, Calif, USA. IL-13 kit was from R&D Systems and TGF-*β*1 from Promega, Madison, Wis, USA.

### 2.12. Lung Histology

Lungs were perfused via the right ventricle with 10 mL of cold PBS, removed, and immersed in 10% phosphate-buffered formalin for 24 h and then in 70% ethanol until embedding in paraffin. Tissues were sliced and 5 *μ*m sections were stained with hematoxylin/periodic acid-Schiff (PAS) for analysis of cellular inflammation and mucus production. 

### 2.13. Statistical Analysis

ANOVA was used to determine the levels of difference between all groups. Comparisons of all pairs were performed by Tukey-Kramer honestly significant difference test. Values for all measurements are expressed as mean ± SEMs, and the *P* values for significance were set to 0.05.

## 3. Results

### 3.1. Oral Tolerance Prevents the Development of Asthma-Like Responses

**Figure 1:**
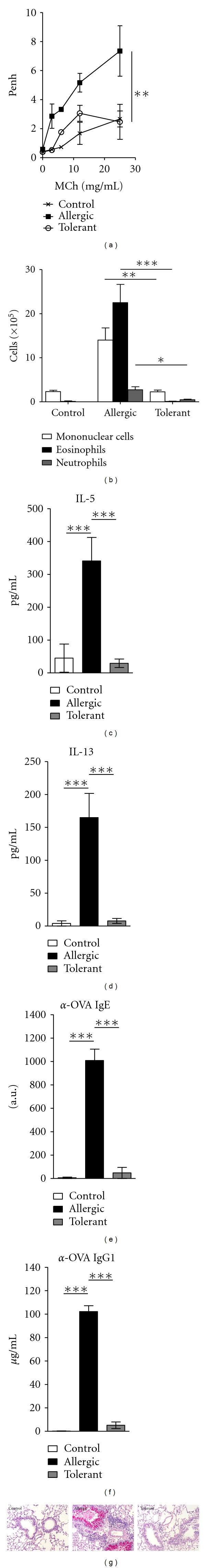
Oral tolerance prevents airway allergic disease. (a) Respiratory pattern to increasing dose of methacholine (MCh) in control, allergic, or tolerant BALB/c mice 24 h after the last OVA challenge. (b) BAL differential cell counts. Quantification by ELISA of (c) IL-5, (d) IL-13 in the BAL fluid, and (e) anti-OVA IgE, (f) IgG1 in the serum. (g) Histology of lung sections at 100x. Lung parenchyma inflammation and mucus production by goblet cells are shown in representative lung sections stained with hematoxylin/PAS. Values represent the means ± SEM for groups of five mice and are representative of more than three experiments. Significant differences *P < 0.05, **P < 0.01, and ***P < 0.001 are shown.

OVA-sensitized and -challenged mice (*Allergic*) developed an enhanced ventilation as revealed by Penh values to increasing doses of methacholine (MCh) compared to untreated mice (*Control*). Conversely, prior oral administration of OVA (*Tolerant*) prevented the increase in ventilation (Figure 1(a)). Differential cell counts showed an increased number of mononuclear cells, neutrophils, and mainly eosinophils in allergic mice compared to control mice. In tolerant mice, the influx of inflammatory cells was almost completely absent (Figure 1(b)). The levels of type-2 cytokines IL-5 and IL-13 in the BAL (Figures 1(c) and 1(d)) and the serum levels of OVA-specific IgE and IgG1 antibodies (Figures 1(e) and 1(f)) were also significantly increased in allergic mice than those orally OVA exposed. Furthermore, lung histology of allergic mice showed intense peribronchial and perivascular inflammatory infiltrates and mucus hypersecretion, determined by PAS staining (Figure 1(g)). In contrast, tolerant mice exhibited lung histology similar to control group (Figure 1(g)). These data show and confirm [12] that OVA-feeding before sensitization efficiently suppresses airway allergic responses and systemic IgE antibody production.

### 3.2. OVA-Feeding Increase Regulatory T Cells in Spleen after Antigen Sensitization

**Figure 2:**
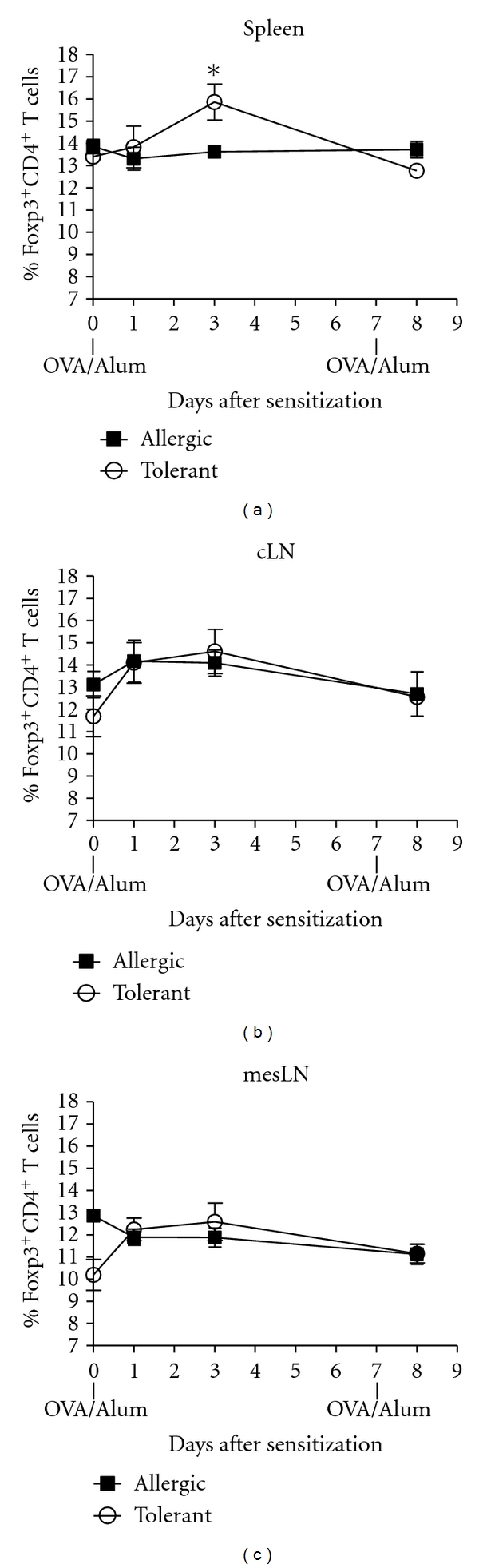
Regulatory T cells in lymphoid organs. Frequency of Foxp3^+^CD4^+^ T cells in (a) spleen, (b) cervical lymph nodes (cLN), and (c) mesenteric lymph nodes (mesLN) of C57BL/6 fed or not with OVA before and after OVA/Alum sensitization. Cells recovered from the different lymphoid organs were stained for CD4 and Foxp3 and gated in CD4-positive cells. Values are representative of two independent experiments with pooled cells from three animals per group.

We and others have previously shown that adaptive CD4^+^CD25^+^ (Foxp3^+^) regulatory T (Treg) cells increase in peripheral lymphoid organs after oral OVA administration in mice with monoclonal OVA-T-cell receptor repertoire [13, 14, 18–23]. Here we were interested in determining whether oral OVA in mice with polyclonal T-cell repertoire could also increase the frequency of Treg cells. For this we monitored the frequency of CD4^+^Foxp3^+^ Treg cells detected in spleen, mesenteric lymph nodes (mesLN), and cervical-draining lymph nodes (cLN) before and after OVA sensitization in mice that received previously OVA or not in the drinking water. We found that the frequency of CD4^+^Foxp3^+^ Treg cells increased at day 3 (d.3) after s.c. OVA sensitization in the spleen of tolerant but not allergic mice and decreased thereafter (Figure 2(a)). No differences were observed between tolerant and allergic mice when the percentages of CD4^+^Foxp3^+^ Treg cells were quantified in cLN and mesLN (Figures 2(b) and 2(c), resp.). These results show that oral OVA administration leads to an increased frequency of spleen Treg cells even in mice with polyclonal T-cell repertoire.

### 3.3. Regulatory T Cells Accumulate in the Airways of Allergic but Not in Tolerant Mice

**Figure 3:**
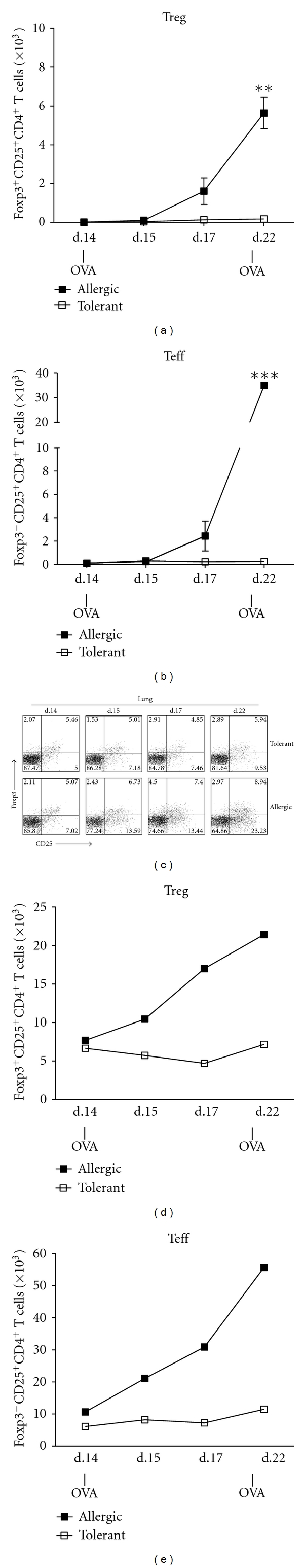
Regulatory T cells accumulate in the airways of allergic but not tolerant mice. Time course of (a) CD4^+^CD25^+^Foxp3^+^ (Treg) and (b) CD4^+^CD25^+^Foxp3^−^ (Teff) cells number in the BAL of allergic and tolerant mice. (c) Frequency and (d) number of CD4^+^CD25^+^ lung cells expressing or not Foxp3. Pooled cells from three mice recovered from BAL and lung were stained for CD4, CD25, and Foxp3 and gated in CD4-positive cells. Values in (a) and (b) represent the means ± SEM for groups of three mice and are representative of two experiments. The data in (c) show a representative experiment of two. Significant differences **P < 0.01, ***P < 0.001 are related to tolerant group.

To monitor the appearance of Treg cells in the airways the number of CD4^+^CD25^+^Foxp3^+^ cells present in the BAL and in the lung tissue were determined from days 14 to 22 (before and after OVA challenges) in mice that received or not OVA in the drinking water. Interestingly, we found an increased number of Foxp3^+^ Treg cells in the BAL of allergic but not tolerant mice. An apparent increase of these cells was found at day 17, that is, 48 h after the first OVA challenge and a significant increase was detected after the second OVA challenge (d.22) (Figure 3(a)). As expected, the number of effector (CD4^+^CD25^+^Foxp3^−^) T (Teff) cells in allergic mice also increased after the first (d.17) and second OVA challenge (Figure 3(b)). Similar results were found in the lungs of allergic group where the frequency and number of both Treg and Teff cells increased after first and second OVA challenge (Figures 3(c), 3(d), and 3(e)). In allergic group at day 22, the number and frequency of Teff cells in the BAL and lung tissue were more than 4-fold higher than Treg cells (Figures 3(a), 3(b), and 3(c)). These results clearly document that Treg cells are recruited at sites of allergen challenge only in mice experiencing allergic inflammation.

### 3.4. Lung Infiltrating Regulatory T Cells Expresses an Effector/Memory Phenotype

**Figure 4:**
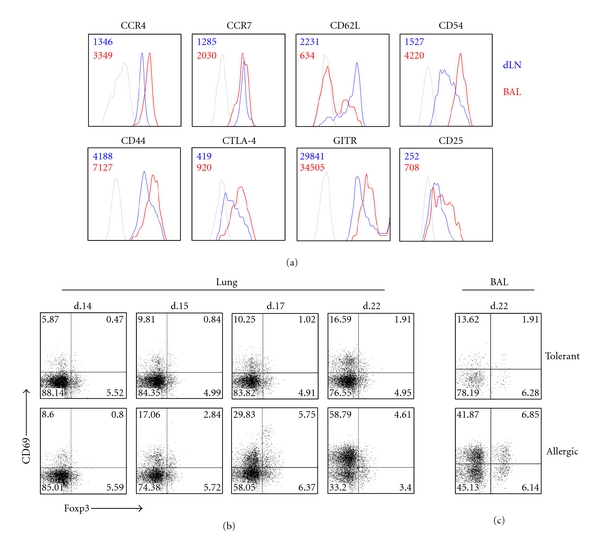
Airway regulatory T cells from allergic mice express a memory/effector phenotype. (a) FACS-Histograms of CD4^+^Foxp3^+^ cells from allergic mice expressing CCR4, CCR7, CD62L, CD54, CD44, CTLA-4, GITR, CD25 in BAL (red line), or mediastinal draining lymph nodes (dLN) (blue line). The numbers into each histogram represent the mean fluorescence intensity (MFI). Kinetic of lung CD4^+^CD69^+^ cells frequency expressing or not Foxp3. (c) Percentage of BAL CD4^+^CD69^+^ cells expressing or not Foxp3. Pooled cells from four mice recovered from lung or BAL were stained for CD4, CD69, and Foxp3 and gated in CD4-positive cells. The results are representative of two experiments with four mice per group.

Because Treg cells were recruited to the airways of allergic mice, we reasoned that these cells might have acquired a migratory phenotype similar to Th2 cells that infiltrate lung tissue [31, 32]. Therefore, we analyzed several T-cell surface molecules associated with T-cell migration and/or activation. As shown in Figure 4(a) by mean fluorescence intensity (MFI) into each FACS-histogram, the BAL CD4^+^Foxp3^+^ Treg cells from allergic mice upregulated the chemokine receptor CCR4 but not CCR7, downregulated L-selectin (CD62L) and upregulated ICAM-1 (CD54) when compared with CD4^+^Foxp3^+^ Treg cells from lung draining lymph nodes (dLN) (Figure 4(a) upper histograms). To further characterize the phenotype of these Treg cells, we determined the expression of activation markers. We found that BAL CD4^+^Foxp3^+^ Treg cells from allergic mice also upregulated CD44, CTLA-4, GITR, and CD25 (Figure 4(a) lower histograms). Moreover, in lung tissue the frequency of CD4^+^Foxp3^+^ Treg cells expressing CD69 molecule increased substantially after OVA challenge in allergic mice compared to tolerant mice (Figure 4(b)). As expected, the frequency of Foxp3-negative CD69^+^ T helper (Teff) cells was drastically enhanced in allergic but not in tolerant group after OVA challenges (Figure 4(b)). Similar results were obtained with T cells present in BAL at day 22 (Figure 4(c)). Notably, the frequency of CD69^+^ Treg cells in the lung and BAL of allergic mice was higher than CD69^−^ Treg cells, whereas in tolerant mice we found an inverse relation (Figure 4(c)). Taken together, our findings clearly indicate that infiltrating Foxp3^+^ Treg cells from allergic mice acquire an effector/memory phenotype distinguishing them from Treg cells present in lung-draining lymph nodes and from those present in the airways of tolerant mice. 

### 3.5. Regulatory T Cells Recruited to the Airways of Allergic Mice Are Not the Principal Producers of Suppressive Cytokines

**Figure 5:**
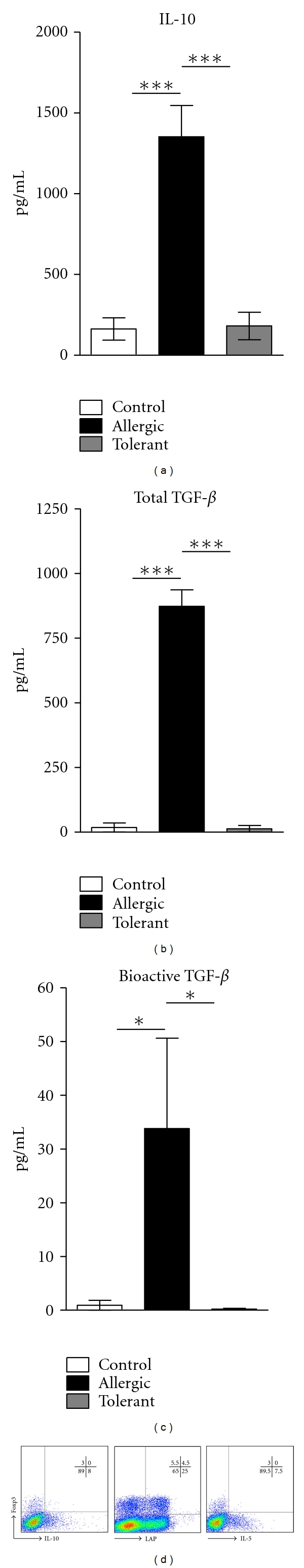
Regulatory T cells are not the major producer of suppressive cytokines in the BAL. Quantification by ELISA of BAL (a) IL-10, (b) total, and (c) bioactive TGF-*β* of BALB/c control, allergic and tolerant mice upon 24 h of the last OVA challenge. (d) IL-10, LAP, and IL-5 staining of BAL CD4^+^Foxp3^+^ cells from allergic mice. Pooled cells recovered from BAL of five allergic mice were stained for CD4, Foxp3, IL-10, LAP, and IL-5 and gated in CD4-positive cells. Values represent the means ± SEM for groups of five mice and are representative of two experiments. Significant differences *P < 0.05, ***P < 0.05 are shown.

Interleukin-10 (IL-10) and transforming growth factor-*β* (TGF-*β*) have been implicated in suppression of inflammation by Treg cells [33–36]. Therefore, we investigated whether airway infiltrating Treg cells from allergic mice produce these cytokines. We first determined the levels of IL-10 and TGF-*β* in BAL fluid. We found that high levels of IL-10, total, and bioactive TGF-*β* were significantly increased in the BAL of allergic mice compared to control or tolerant groups (Figures 5(a), 5(b), and 5(c)). To ascertain whether Treg cells of allergic mice produce these suppressive cytokines, we stained CD4^+^Foxp3^+^ T cells for intracellular IL-10 or for latent-associated peptide (LAP) to indirectly detect TGF-*β* producing cells. TGF-*β* complexes with latency-associated peptide (LAP), and LAP expression correlates with TGF-*β* production in many cell types [37–39]. We found that only CD4^+^Foxp3^−^ cells stained positively for IL-10. The expression of LAP was found in both Foxp3^−^ and Foxp3^+^ cells, however, the majority (25%) of CD4^+^ cells in the BAL expressing LAP were Foxp3^−^ (Figure 5(d)). As expected, Foxp3^+^ T cells did not produce IL-5 (Figure 5(d)). These results indicate that high levels of suppressive cytokines at site of allergen challenge are associated with lung allergic inflammation and that CD4^+^Foxp3^+^ Treg cells in the airways of allergic mice do not produce IL-10 and are not the major population of TGF-*β* producing cells. 

### 3.6. Lung Treg Cells of Allergic Mice Exhibit Strong Antiproliferative Activity but Are Unable to Suppress Type-2 Cytokine Production

**Figure 6:**
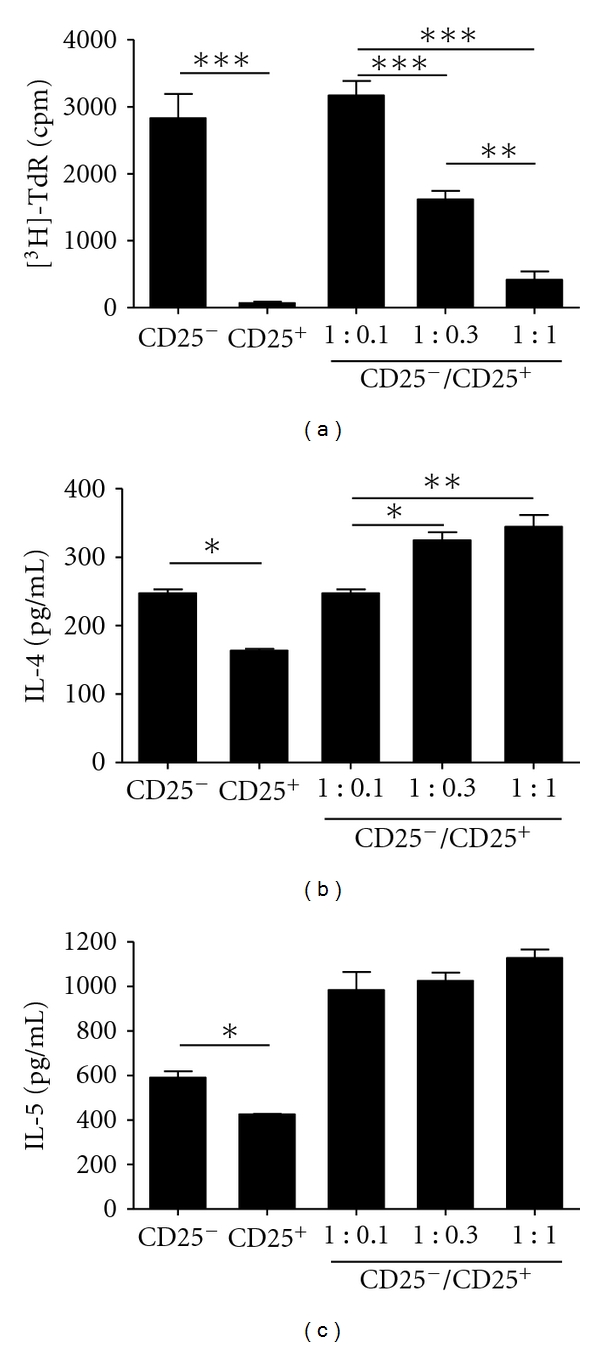
Lung CD4^+^CD25^+^ T cells from allergic mice suppress T-cell proliferation but not Th2 cytokine production. (a) Proliferation of MACS-purified lung CD4^+^CD25^−^ or CD4^+^CD25^+^ cells from BALB/c allergic mice alone or cocultured at different CD25^−^/CD25^+^ ratios determined by ^3^H-thymidine (^3^H-TdR) incorporation after anti-CD3 stimulation. ELISA assays for (b) IL-4 and (c) IL-5 in the culture supernatants showed in (a). Values represent the means ± SEM of triplicate wells. The results are representative of two experiments. Significant differences *P < 0.05, **P < 0.01, and ***P < 0.001 are shown.

**Figure 7:**
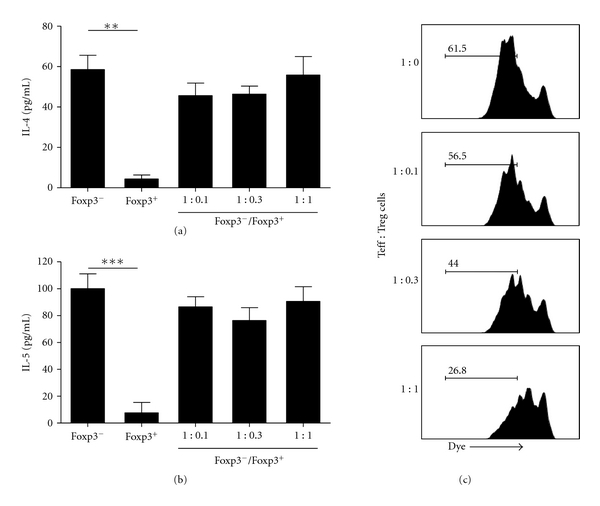
Lung Foxp3^+^ Treg cells from allergic mice suppress T-cell proliferation but not type 2 cytokine production. (a) IL-4 and (b) IL-5 levels in the supernatants of FACS-sorted lung CD4^+^Foxp3-GFP^+^ (Foxp3^+^) or CD4^+^Foxp3-GFP^−^ (Foxp3^−^) cells from *Foxp3gfp.*KI allergic mice alone or cocultured at different Foxp3^−^/Foxp3^+^ ratios. (c) Proliferation of Foxp3-GFP^−^ cells cocultured at different Foxp3^−^/Foxp3^+^ ratios was determined by flow cytometry. Sorted lung CD4^+^Foxp3-GFP^−^ were labeled with Dye eFluor-670 and the proliferation was determined by reduction of the fluorescence intensity of Dye. Representative FACS-histograms, which indicate the frequency of T-cell proliferation, are shown. Values represent the means ± SEM of triplicate wells. The results are representative of two experiments. Significant differences **P < 0.01, ***P < 0.001 are shown.

Finally, to address the role of Treg cells present in the lung of allergic mice, we performed a standard *in vitro* suppression assay [40, 41], as previously described [24]. First, we tested the proliferative activity of CD4^+^CD25^−^ (memory/effector T cells) cells from lung upon anti-CD3 stimulation in the presence or absence of CD4^+^CD25^+^ cells. As shown in Figure 6(a), lung CD4^+^CD25^−^ cells exhibited high proliferative response upon anti-CD3 antibody stimulation whereas lung CD4^+^CD25^+^ cells did not proliferate. Coculture of CD4^+^CD25^+^ with CD4^+^CD25^−^ cells showed that CD25^+^ cells almost completely suppressed CD25^−^ cell proliferation at ratio 1 : 1, partially at 0.3 : 1 but not at 0.1 : 1 (Figure 6(a)). Next, we evaluated the production of Th2-cytokine by lung CD4^+^CD25^−^ cells in the presence or absence of CD4^+^CD25^+^ cells. Albeit CD4^+^CD25^+^ cells efficiently suppressed T-cell proliferation, they were unable to inhibit IL-4 and IL-5 production upon anti-CD3 stimulation (Figures 6(b) and 6(c)). Similar data were found when these cells were stimulated specifically with OVA (data not shown). The lack of inhibition of IL-4 and IL-5 secretion by CD4^+^CD25^+^ cells might be due to the fact that this cell population also contains effector T cells. Indeed, CD4^+^CD25^+^ cells produced significant amounts of type-2 cytokines (Figures 6(b) and 6(c)). In order to circumvent this problem and address more directly whether Treg cells affect type-2 cytokine production, we performed experiments in *Foxp3gfp.*KI mice that harbor fluorescent Treg cells [29]. Therefore, we induced airway allergic disease in *Foxp3gfp.*KI mice and sorted CD4^+^ T cells expressing Foxp3-GFP^+^ Treg cells and CD4^+^GFP^−^ T cells (Foxp3^−^) present in the lungs. We found that only Foxp3^−^ T cells produced significant amounts of type 2 cytokines upon anti-CD3 stimulation (Figures 7(a) and 7(b)). Notably, a highly purified (>98%) lung population of Foxp3-GFP^+^ Treg cells could not suppress efficiently Th2 cytokine production by CD4^+^Foxp3-GFP^−^ T cells upon anti-CD3 stimulation (Figures 7(a) and 7(b)). Finally, through using purified lung Foxp3-GFP^+^ Treg cells, we confirmed the suppression assay obtained with CD4^+^CD25^+^ T-cell by showing that they efficiently suppressed T effector (CD4^+^GFP^−^) cells proliferation at ratio 1 : 1 and 0.3 : 1 but not at 0.1 : 1 as evidenced by Dye eFluor-670 staining (Figure 7(c)). We conclude that lung Treg cells with regulatory phenotype present in the airways of allergic mice exhibit a strong antiproliferative activity but are unable to efficiently suppress type-2 cytokine production.

## 4. Discussion

A critical issue in immune regulation is where Treg cells exert their suppressive function. Their presence on lymphoid tissue appears to be required for efficient suppression of naive T-cell activation. Conversely, some data indicate that Treg cells are recruited to effector site in order to suppress the action of inflammatory T cells [25, 42, 43]. Previous reports showed a relationship between suppression of asthma-like responses by mucosal tolerance and the emergence of Treg cells in lymphoid organs [17, 21, 24]. We have previously shown in T/B receptors transgenic mice (T-Bmc) devoid of natural regulatory T cells that soon after mucosal antigen exposure, Foxp3-expressing Treg cells are generated in dLN and in spleen [24]. This early induction of Treg cells by prior oral antigen exposure appears to inhibit the development of polarized Th2 inflammatory cells in a TGF-*β*-dependent manner [24]. Indeed, using the T-Bmc model, we found that Treg cells are able to suppress early T-cell activation, 48 h after immunization with the cognate antigen [24]. However, after establishment of tolerance they became dispensable for its maintenance* in situ*. In the present study, we used a well-established model of mucosal tolerance to allergic lung inflammation [8, 12, 24, 44] to monitor the appearance of Treg cells in the airways after OVA challenge in mice with polyclonal repertoire. We found that only in OVA-fed mice, the frequency of spleen Treg cells increased at day 3 after OVA sensitization, a result resembles the T-Bmc model. However, here we were particularly interested in determining whether Treg cells migrate to airways of allergic or tolerant mice after administration of OVA. We found that allergic but not tolerant mice showed a striking increase in the number of Treg cells in the BAL compared to tolerant mice. Also, high levels of IL-10 and TGF-*β* were detected in the airways of allergic mice. Notably, we found that among CD4^+^ T cells recruited to allergic inflammation only Foxp3-negative, but not Foxp3-positive T cells stained positively for IL-10. Moreover, the majority of LAP^+^ cells were Foxp3-negative T cells. Our results are line with data obtained with T-cell infiltrates in *Shcistosoma mansoni *egg-induced Th2-mediated inflammation [45]. In concert with our findings, migration of Treg cells was also reported in a model of parasite egg antigens-induced inflammation [46], or other pathological conditions, such as arthritis, type 1 diabetes, sarcoidosis and transplants [25, 43, 47–52]. Therefore, it is plausible that the allergic inflammatory milieu triggers the migration of Treg cells into the airways. Accordingly, it has been shown that recruitment of Foxp3-expressing Treg cells to the site of allergic inflammation is dependent on chemokine receptors such as CCR4 [52] and CCR8 [53], where their ligands CCL17, CCL22, and CCL1 are high expressed during allergic lung inflammation [54, 55]. Our data demonstrated that the majority of Foxp3-expressing Treg cells present in the airways upregulated CCR4, CD44 and CD54 and drastically downregulated CD62L, a phenotype that resembles effector/memory T cells. Noteworthy, this phenotype could distinguish Treg cells present in the airways from those present in the lung-draining lymph nodes (dLN). In addition, we showed that Treg cells that accumulated in the airways of allergic mice also acquired activated phenotype, as revealed by increased expression of CTLA-4, GITR, and CD25 contrasting with Treg cells present in the dLN. Moreover, CD69, a marker of cell activation, was highly expressed in Treg cells present in the lung and BAL of allergic mice but not in tolerant group. These data suggest a functionally important activation step that accompanies Treg cell migration. The loss of CD62L and the increased of CD54 expression by Treg cells could also contribute to their migration to the lung [32]. A picture that emerges from our findings is that Treg cells get activated and are recruited to sites of allergic inflammation probably because at these sites CCR4-specific ligands are expressed at high levels [28, 56]. 

It was recently reported that the loss of CCR4 severely inhibited the accumulation of CD4^+^CD25^+^ T cells in the lung and skin [57]. CCR4 knockout mice also fail to develop allograft tolerance after administration of anti-CD154 with donor spleen cells, which is associated with a decreased of Foxp3^+^ T cells in the graft [43]. Previous data indicated a division of labor between naive and activated Treg cells [58]. For instance, naive-like Treg cells use the chemokine receptor CCR7 for recirculation through lymph nodes where they control the priming phase of an immune response whereas CCR7 is dispensable in effector/memory-like Treg cells for their accumulation in inflamed sites and in fact CCR7-deficiency enhance Treg cells-mediated suppression of inflammation [58]. In our model, the role of CCR7 could not be established because activated lung Treg cells expressed similar levels of CCR7 when compared to naive dLN Treg cells [25]. Using an islet allograft model it was demonstrated that Treg cells first migrate from blood to the allograft where they become activated, and then they migrate to the dLN in a CCR7 fashion. This movement was essential for optimal suppression allograft rejection [25]. A similar situation was found by Graca et al. that found regulatory T cells in skin allografts suggesting that T-cell suppression of graft rejection is an active process that involves the presence of regulatory T cells at the site of the tolerated transplant [59]. This scenario does not appear to operate in our model because we did not find Treg cells in dLN with an activated phenotype. 

We first studied the suppressive activity of airway CD4^+^CD25^+^ T cells, putative Treg cells, in order to determine their role in lung inflammation. We clearly showed that CD4^+^CD25^+^ T cells containing activated Foxp3^+^ Treg cells efficiently suppressed the proliferation of lung CD4^+^CD25^−^ memory/effector T cells. Strikingly, these CD4^+^CD25^+^ T cells did not suppress the secretion of IL-4 and IL-5 by anti-CD3 or OVA-activated CD4^+^CD25^−^ T cells. Because CD4^+^CD25^+^ T cells contain also effector Foxp3-negative T cells, it is likely that these cells were the source of the type 2 cytokines detected in the cultures. To circumvent this we purified lung fluorescent Foxp3 Treg cells from allergic *Foxp3gfp.*KI mice and tested their suppressive activity on type 2 cytokine production by Foxp3-negative CD4^+^ T cells. In this situation, Foxp3-positive Treg cells did not secrete type 2 cytokines and did not suppress significantly type 2 cytokine production by Foxp3-negative CD4^+^ T cells but did suppress CD4^+^ T-cell proliferation. These results could explain why, despite the large infiltration of Treg cells, allergic mice still show Th2-associated pathological responses. Our results are in line with previous finding showing that in allergic patients, CD4^+^CD25^+^ T cells did not suppress the release of Th2 cytokines [6]. The inefficiency of Treg cell in suppressing inflammatory cytokines in established pathological conditions was also reported in sarcoid granulomas, in which Treg cells suppressed T-cell proliferation but were unable to inhibit TNF-*α* secretion [51]. Notably, in a model of autoimmune encephalomyelitis, Treg cells also expand after exposure to myelin antigens and infiltrate the central nervous system, but these infiltrating Treg cells were unable to suppress proliferation and inflammatory cytokine production of effector T cells from target tissue [60]. Based on our results and previous reports, it appears that the inflammatory milieu impairs Treg-cell functions.

In summary, we showed that oral tolerance was not associated with an increased number of Treg cells or suppressive cytokines in the airways. Conversely, allergic inflammation triggers the infiltration of Treg cells into the airways that efficiently suppress T-cell proliferation but not Th2 cytokine production. Our findings suggest that allergic inflammation renders the suppressive activity of Treg cells less stringent that, in turn, allows the manifestations allergic reactions mediated by type 2 cytokines.
